# Neuropilin 1 Mediates Keratinocyte Growth Factor Signaling in Adipose-Derived Stem Cells: Potential Involvement in Adipogenesis

**DOI:** 10.1155/2018/1075156

**Published:** 2018-02-25

**Authors:** Simona Ceccarelli, Cristina Nodale, Enrica Vescarelli, Paola Pontecorvi, Valeria Manganelli, Giovanni Casella, Maria Giuseppina Onesti, Maurizio Sorice, Ferdinando Romano, Antonio Angeloni, Cinzia Marchese

**Affiliations:** ^1^Department of Experimental Medicine, Sapienza University of Rome, Viale Regina Elena 324, 00161 Roma, Italy; ^2^Department of Surgical Sciences, Sapienza University of Rome, Viale Regina Elena 324, 00161 Roma, Italy; ^3^Department of Surgery “P. Valdoni”, Sapienza University of Rome, Viale del Policlinico 155, 00161 Roma, Italy; ^4^Department of Public Health and Infectious Diseases, Sapienza University of Rome, Piazzale Aldo Moro 5, 00185 Roma, Italy

## Abstract

Adipogenesis is regulated by a complex network of molecules, including fibroblast growth factors. Keratinocyte growth factor (KGF) has been previously reported to promote proliferation on rat preadipocytes, although the expression of its specific receptor, FGFR2-IIIb/KGFR, is not actually detected in mesenchymal cells. Here, we demonstrate that human adipose-derived stem cells (ASCs) show increased expression of KGF during adipogenic differentiation, especially in the early steps. Moreover, KGF is able to induce transient activation of ERK and p38 MAPK pathways in these cells. Furthermore, KGF promotes ASC differentiation and supports the activation of differentiation pathways, such as those of PI3K/Akt and the retinoblastoma protein (Rb). Notably, we observed only a low amount of FGFR2-IIIb in ASCs, which seems not to be responsible for KGF activity. Here, we demonstrate for the first time that Neuropilin 1 (NRP1), a transmembrane glycoprotein expressed in ASCs acting as a coreceptor for some growth factors, is responsible for KGF-dependent pathway activation in these cells. Our study contributes to clarify the molecular bases of human adipogenesis, demonstrating a role of KGF in the early steps of this process, and points out a role of NRP1 as a previously unknown mediator of KGF action in ASCs.

## 1. Introduction

Adipose-derived mesenchymal stem cells (ASCs) represent a population of self-renewing and multipotent cells that reside in the vascular stroma of adipose tissue and, when appropriately stimulated, can differentiate into several cell types, that is, adipocytes, myocytes, chondrocytes, and osteocytes [1]. These cells play important roles in development, post-natal growth, tissue repair, and regeneration [2, 3]. Based on these properties, ASCs are a powerful tool not only for regenerative cell-based therapy but also for investigating the molecular mechanism involved in adipogenesis.

Adipogenic differentiation has been shown to be regulated by a complex network of transcription factors, cofactors, and signaling intermediates from numerous pathways that begins with the transient expression of CCAAT/enhancer binding protein *β* (CEBP*β*) and CEBP*δ*, which in turn activate peroxisome proliferator-activated receptor *γ* (PPAR*γ*) and CEBP*α* [4].

Several members of the fibroblast growth factor (FGF) family have been reported to regulate adipogenesis. In particular, FGF1 is produced in adipose tissue and acts through its specific receptor FGFR1 to enhance proliferation, commitment, and differentiation of preadipocytes [5–7]. FGF2 and FGF10 are also expressed in adipose tissue and have each been implicated in adipogenesis. FGF2 is regulated through adipogenic differentiation and displays concentration-dependent biphasic effects on the expression of adipogenic genes [8, 9]. FGF10 is essential for the development of adipose tissue, since on the one hand it stimulates the proliferation of preadipocytes through the activation of RAS/MAPK pathway [10–12] and on the other hand it regulates adipogenic differentiation by contributing to the expression of adipogenic genes such as CEBP*β* and PPAR*γ* [13].

KGF, another member of FGFs family, also known as FGF7, has been also detected in adipose tissue [14, 15]. KGF is produced by cells of mesenchymal origin and mainly acts in a paracrine way on epithelial cells, playing an important role in organogenesis, vasculogenesis, and regeneration of different organs [16, 17] as well as in cellular processes such as proliferation and migration [18, 19]. It is known that KGF acts by binding to the FGFR2-IIIb receptor, also called KGFR, which is generally expressed in epithelial cells. Its alternatively spliced isoform FGFR2-IIIc, predominantly expressed in cells of the mesenchymal lineage, usually binds other members of the FGF family, such as FGF1 and FGF2 [20]. Nevertheless, recently it has been reported that KGF is able to promote proliferation of murine preadipocytes through the activation of the PI3K-Akt signaling pathway [21]. In addition, KGF seems to stimulate the expression of key regulators of adipogenesis, such as CEBP*α*, CEBP*δ*, and sterol-regulatory element binding protein-1 (SREBP-1), in alveolar type II cells [22]. However, KGF effect on human ASCs has not been yet investigated.

Moreover, although KGF has been reported to contribute to preadipocyte proliferation and adipogenesis in an autocrine manner, the involvement of FGFR2-IIIb in its action has not been demonstrated, and previous works suggested that KGF stimulation of preadipocyte proliferation might happen through an unknown receptor rather than FGFR2-IIIb [21].

Neuropilin 1 (NRP1) is a transmembrane glycoprotein that serves as a receptor for various types of ligands, such as the class 3 semaphorins in neurons, the vascular endothelial growth factor (VEGF) family in endothelial cells, and the platelet-derived growth factor (PDGF) in megakaryocytes [23, 24]. NRP1, although generally considered a coreceptor for these growth factors, has been observed to induce activation of some signaling pathways also independently of other tyrosine kinase receptor expression [25].

Our study has been focused to better define the potential role of KGF in human adipogenesis by analyzing its expression during adipogenic differentiation, and in adipose-derived stem cell proliferation by assessing its effect on the activation of cellular signaling in ASCs. Then, we analyzed its mechanism of action, trying to determine the receptor involved in KGF-mediated stimulation of ASCs.

This work could contribute to a better understanding of the molecular bases of adipogenesis, and it will also highlight a previously unknown role of Neuropilin 1 as a mediator of KGF action in these cells.

## 2. Materials and Methods

### 2.1. Adipose Tissue Donors

Subcutaneous adipose tissue samples were harvested from abdominal wall resections of seven healthy donors (age range: 22–55 years, BMI range: 18.5–24.9 kg/m^2^), who underwent elective plastic surgery. Donors had no systemic diseases or diabetes, and neither took drugs that could affect the mass of adipose tissue and metabolism. The deidentified samples were transported to the laboratory and processed within 24 h of collection.

The use of clinical samples of adipose tissue for ASC isolation complied with the Declaration of Helsinki 1975, revised in 2008, and has been approved by the Institutional Review Board of the Department of Experimental Medicine of the Sapienza University of Rome (Ref. 11.1.2/27-10-2015). Written consent was obtained from all subjects.

### 2.2. Cell Isolation and Culture

The adipose tissue samples were transferred into a sterile tube and washed extensively with sterile phosphate-buffered saline (PBS) containing 2% PSG to remove contaminating debris and red blood cells. Then, adipose tissue was transferred into a Petri dish and digested with 0.075% collagenase (type I; Gibco, Paisley, UK) in PBS at 37°C for 30–60 min, with gentle agitation, to break down the extracellular matrix. The collagenase was inactivated with an equal volume of Dulbecco's modified Eagle's medium (DMEM; Gibco) supplemented with 10% fetal bovine serum (FBS), and the suspension was gently pipetted until disintegration and filtered through a 100 *μ*m mesh filter to remove debris. The stromal-vascular fraction (SVF), which contains ASCs, was then pelleted by centrifugation for 5 min at 2000 rpm. The pelleted cells were resuspended in DMEM-Ham's F-12 medium (vol/vol, 1 : 1) (DMEM/F12; Gibco) supplemented with 20% FBS, 100 U/ml penicillin, 100 mg/ml streptomycin, and 2 mM L-glutamine and plated in a 75 cm^2^ tissue culture flask coated with collagen (type IV; Sigma-Aldrich, Milan, Italy). The SVF fraction contains an unpurified population of stromal cells, which includes ASCs, but also circulating cell types, hematopoietic stem cells, and endothelial progenitor cells. ASCs were self-selected out of the SVF during subsequent tissue culture passages, since they are adherent to the plastic tissue cultureware.

ASCs were maintained in a 5% CO_2_ incubator at 37°C in a humidified atmosphere, with medium change twice a week. When reaching 80–90% confluence, cells were detached with 0.5 mM EDTA/0.05% trypsin (Gibco) for 5 min at 37°C and replated. ASCs were expanded and cell viability was assessed by using the trypan blue exclusion assay. Cell morphology was evaluated by phase contrast microscopy. A homogeneous population of ASCs was subsequently checked by analyzing the surface marker expression profile. For all the experiments, ASCs were used between passages 2 and 6.

The adenocarcinoma cell lines MCF-7 and MDA-MB-231 were obtained from the American Type Culture Collection (ATCC-LGC Promochem, Teddington, UK) and cultured in Dulbecco's Modified Eagle's Medium (DMEM; Invitrogen, Karlsruhe, Germany), supplemented with 10% fetal bovine serum (FBS; Invitrogen) and antibiotics. Primary cultures of human fibroblasts (HFs) were established from 1 cm^2^ full-thickness skin biopsy from a healthy donor, as previously described [26], and maintained in DMEM containing 10% FBS.

### 2.3. Flow Cytometry

Flow cytometric analyses were performed by using a FACSCalibur cytometer (BD Biosciences, San Jose, CA, USA), as previously described [1]. Briefly, cultured ASCs were harvested, centrifuged, and fixed for 30 min in ice-cold 2% paraformaldehyde. The single-cell suspensions were washed in flow cytometry buffer containing PBS, 2% FBS, and 0.2% Tween 20 and then incubated for 30 min with monoclonal antibodies to CD29, CD34, CD44, CD45, CD90, and CD166, conjugated to fluorescein isothiocyanate, phycoerythrin, or phycoerythrin-Cy5 (BD Biosciences). All monoclonal antibodies were of the IgG1 isotype. Nonspecific fluorescence was determined by incubating the cells with conjugated mAb anti-human IgG1 (DakoCytomation, Glostrup, Denmark).

### 2.4. Multilineage Differentiation

The adipogenic, chondrogenic, and osteogenic differentiation of cultured ASCs was achieved using the Human Mesenchymal Stem Cell Functional Identification Kit (R&D Systems, Inc., Minneapolis, MN, USA), which contains specially formulated media supplements and a panel of antibodies to define the mature phenotypes of adipocytes, chondrocytes, and osteocytes, as previously reported [27]. In particular, for adipogenic differentiation, cells were resuspended in *α*MEM (Gibco) supplemented with 10% FBS, 100 U/ml penicillin, 100 *μ*g/ml streptomycin, and 2 mM L-glutamine and seeded on coverslips onto 24-well plates, at a density of approximately 3.7 × 10^4^ cells/well. Medium was replaced every 2-3 days until 100% confluency was reached, then cells were incubated with adipogenic differentiation medium (ADM, *α*MEM supplemented with a solution containing hydrocortisone, isobutylmethylxanthine, and indomethacin; R&D Systems), to induce adipogenesis. In KGF-treated cells, ADM was supplemented with 1 ng/ml KGF (Upstate Biotechnology, Lake Placid, NY). The ADM or ADM + KGF was replaced every 3-4 days. The appearance of lipid vacuoles was monitored by microscopic examination.

### 2.5. Immunofluorescence Analysis

Immunofluorescence was performed as previously described [28]. Briefly, cells grown on coverslips onto 24-well plates were fixed in 4% paraformaldehyde for 30 min at room temperature, followed by treatment with 0.1 M glycine in PBS for 20 min and with 0.1% Triton X-100 in PBS for additional 5 min to allow permeabilization. Cells were then incubated with the following primary antibodies: anti-human CD29 monoclonal antibody, anti-human CD166 monoclonal antibody (BioLegend, San Diego, CA, USA), anti-human CD34 monoclonal antibody (BD Biosciences), and goat anti-mouse FABP4 antibody (R&D Systems). After appropriate washing in PBS, primary antibodies were visualized using FITC-conjugated goat anti-mouse IgG (Cappel Research Products, Durham, NC, USA) or Texas Red-conjugated rabbit anti-goat IgG (Jackson ImmunoResearch Laboratories, West Grove, PA, USA).

For all immunofluorescence experiments, nonspecific fluorescence was determined by omitting primary antibody. Nuclei were visualized using 4′,6-diamidino-2-phenylindole (DAPI) dihydrochloride (Sigma-Aldrich). The single-stained and merged images were acquired with AxioVision software (Carl Zeiss, Jena, Germany) using a 20x objective lens. Quantitative analysis was performed by counting cells exhibiting FABP4 expression (expressed as percentage of FABP4-positive cells). More than 50 stained cells per microscopic field were counted. Results from three microscopic fields, expressed as mean ± standard deviation, were reported in graphs.

### 2.6. Oil Red O Stain

After 21 days from induction with ADM or ADM + KGF, cells were fixed in 10% formalin for 30–60 minutes at room temperature, incubated in 60% isopropanol for 5 minutes and stained with oil red O solution, which identifies lipids, for 5 minutes. The images were acquired with AxioVision software (Carl Zeiss, Jena, Germany) using a 20x objective lens. Subsequently, the stained oil droplets were treated with isopropanol to elute oil red O dye, and the absorbance was quantified at 490 nm. Results from three independent experiments, expressed as mean ± standard deviation, were reported in a graph.

### 2.7. MTT Assay

Cells were plated onto 96-well plates at a density of 2.5 × 10^3^ cells/well in DMEM-Ham's F-12 supplemented with 10% FBS, then serum starved for 18 h and treated or not with 20 ng/ml KGF for 1–5 days. Then, 0.5 mg/ml MTT (3-(4,5-dimethylthiazol-2-yl)-2,5-diphenyltetrazolium bromide; Sigma, St. Louis, MO, USA) was added, mixed into each well, and incubated at 37°C. After 4 h, the MTT-medium mixture was removed and 100 *μ*l dimethyl sulphoxide (DMSO; Sigma, St. Louis, MO, USA) was added in each well. The absorbance value was measured at 490 nm with an ELISA Microplate Reader (Bio-Rad, Hercules, CA, USA). Results from three independent experiments, expressed as mean ± standard deviation, were reported in a graph.

### 2.8. RT-PCR and Quantitative Real-Time PCR (qRT-PCR)

ASCs were harvested and total RNA was extracted with the use of TRIzol reagent (Invitrogen). cDNA was generated with oligo (dT) from 1 *μ*g of RNA using the SuperScript III Reverse Transcriptase Kit (Invitrogen). Specific NRP1 PCR primers (Table 1) were used, and PCR conditions were as follows: hold for 2 min at 94°C, followed by 40 cycles consisting of denaturation at 94°C (15 s), annealing at 62°C (30 s), and elongation at 72°C (20 s). The amplified products were subjected to electrophoresis in a 1.5% agarose gel. GAPDH was used as a reference gene. Quantitative real-time PCR assays (qRT-PCR) were conducted in triplicate on an ABI 7500 Real Time instrument (Applied Biosystems) as previously described [29]. Briefly, the abundance of KGF, FABP4, or NRP1 was quantified using the appropriate real-time TaqMan gene expression assay kit (Applied Biosystems by Life Technologies, Carlsbad, CA, USA). Cyclophilin A (PPIA) mRNA was used as endogenous control. For FGFR2-IIIb and FGFR2-IIIc, specific custom TaqMan primer/probe assays were developed and used at a concentration of 1x per well. Primers and probes are reported in Table 1. The absolute copy number of the target transcripts was obtained according to a plasmid DNA standard curve, as previously described [30]. The analysis of the adipogenic differentiation marker PPAR*γ* was performed using SYBR Green PCR master mix kit (Applied Biosystems), and primers were designed according to Newell et al. [6].

### 2.9. Immunoprecipitation and Western Blot Analysis

Cells, treated or not with 20 ng/ml KGF for the indicated times, were lysed in RIPA buffer. For immunoprecipitation experiments, 800 *μ*g of lysates were incubated with anti-NRP1 antibody overnight at 4°C, and then for 2 h at 4°C with 20 *μ*l Protein A/G PLUS-Agarose (Santa Cruz). Immune complexes were collected by centrifugation and washed three times with ice-cold RIPA buffer. After a final wash, the supernatant was discarded and the pellet was resuspended in SDS lysis buffer, and then boiled in 4x SDS loading dye for 5 min. Proteins were resolved under reducing conditions by 10% SDS-PAGE and transferred to Immobilon-FL membranes (Merck Millipore, Billerica, MA, USA). Membranes were blocked in TBS containing 0.1% Tween 20 (TBS-T) and 5% milk for 1 h at 25°C and then incubated overnight at 4°C with the following primary antibodies: anti-Neuropilin 1 (A-12) and anti-FGF7 (Santa Cruz Biotechnology, Santa Cruz, CA, USA). Membranes were then incubated with a goat anti-mouse IgG1 heavy chain- (HRP) conjugated secondary antibody (Abcam, Cambridge, UK) for 1 h at 25°C. Bound antibody was detected by enhanced chemiluminescence detection reagents (Pierce Biotechnology Inc., Rockford, IL, USA), according to the manufacturer's instructions. For Western blot analysis, total proteins (50–150 *μ*g) were resolved under reducing conditions by 7–10% SDS-PAGE and transferred to Immobilon-FL membranes (Merck Millipore, Billerica, MA, USA), as previously described [28]. Membranes were blocked in TBS containing 0.1% Tween 20 (TBS-T) and 5% milk for 1 h at 25°C and then incubated overnight at 4°C with the following primary antibodies: anti-phospho-p44/42 MAPK (Thr202/Tyr204), anti-phospho-Akt (Ser473), anti-Akt, anti-phospho-p38 MAPK (Thr180/Tyr182), anti-p38 MAPK, anti-phospho-Rb (Ser807/811), anti-Rb (Cell Signaling Technology Inc., Danvers, MA, USA), anti-ERK2, anti-Bek (C-17), anti-Neuropilin 1 (A-12), anti-FGF7 (Santa Cruz Biotechnology, Santa Cruz, CA, USA), and anti-*β*-tubulin (Sigma-Aldrich). FGFR2-IIIb expression was achieved using a homemade mouse monoclonal antibody raised against a peptide corresponding to amino acids 314–361 of the human FGFR2-IIIb [31].

Membranes were then incubated with the appropriate horseradish peroxidase- (HRP-) conjugated secondary antibody (Santa Cruz Biotechnology) for 1 h at 25°C. Bound antibody was detected by enhanced chemiluminescence detection reagents (Pierce Biotechnology Inc., Rockford, IL, USA), according to the manufacturer's instructions. Tubulin served to estimate the protein equal loading. Densitometric analysis was performed using Quantity One Program (Bio-Rad Laboratories S.r.l., Segrate, MI, Italy).

### 2.10. High-Performance Thin Layer Chromatography (HPTLC)

ASCs untreated or treated with 20 ng/ml KGF for 24 h were lysed in lysis buffer containing 1% Triton X-100, 10 mM TRIS-HCl (pH 7.5), 150 mM NaCl, 5 mM EDTA, 1 mM Na_3_VO_4_, and 75 U of aprotinin and allowed to stand for 20 min at 4°C. The cell suspension was mechanically disrupted by Dounce homogenization (10 strokes). Then, lysate was centrifuged for 5 min at 1300 ×g to remove nuclei and large cellular debris. After evaluation of the protein concentration by Bradford Dye Reagent assay (Bio-Rad, 500-0006), the lysate was subjected to lipid analysis. Neutral lipid extracts were separated by high-performance thin layer chromatography (HPTLC) using a solvent system of hexane/diethyl ether/acetic acid (70 : 30 : 1, *v*/*v*/*v*) and were detected by staining with 2% copper acetate solution in 8% phosphoric acid and subsequent heating at 120°C for 15 min. After about 3 min, free cholesterol, cholesterol esters, and triglycerides yielded red spots on a white background, which were converted into pink-brown spots after 10 min. Quantitative analysis was carried out using NIH Image1.62 as software (Mac OS X, Apple Computer International).

### 2.11. siRNA-Mediated Downregulation of FGFR2 and NRP1

The FGFR2-specific (siBek) or NRP1-specific (siNRP) short interfering RNA, which specifically knock down FGFR2 and Neuropilin 1 gene expression, respectively, as well as negative control siRNA (siNC), which does not lead to the specific degradation of any cellular mRNA, were purchased from Santa Cruz Biotechnology. ASCs were transfected with siRNA at a final concentration of 25 nM, using the HiPerFect Transfection Reagent (Qiagen, Valencia, CA, USA) according to the manufacturer's instructions. Transfected cells were incubated at 37°C for 48 h before starvation, treated with 20 ng/ml KGF for 5 min at 37°C, and processed for Western blot assay. The reduction of FGFR2 or NRP1 expression in ASCs was confirmed by immunoblotting.

### 2.12. Statistical Analysis

Data were analyzed using one-way analysis of variance (ANOVA) after Bartlett's test for the homogeneity of variances and Kolmogorov-Smirnov's test for the Gaussian distribution and followed by Newman-Keuls multiple-comparison test or, when appropriate, with Student's *t*-test for paired samples assuming a two-tailed distribution. All data reported were verified in at least three different experiments and reported as mean ± standard deviation (SD). Only *P* values < 0.05 were considered as statistically significant.

## 3. Results

### 3.1. Phenotypic Characterization of ASC Cultures

**Figure 1:**
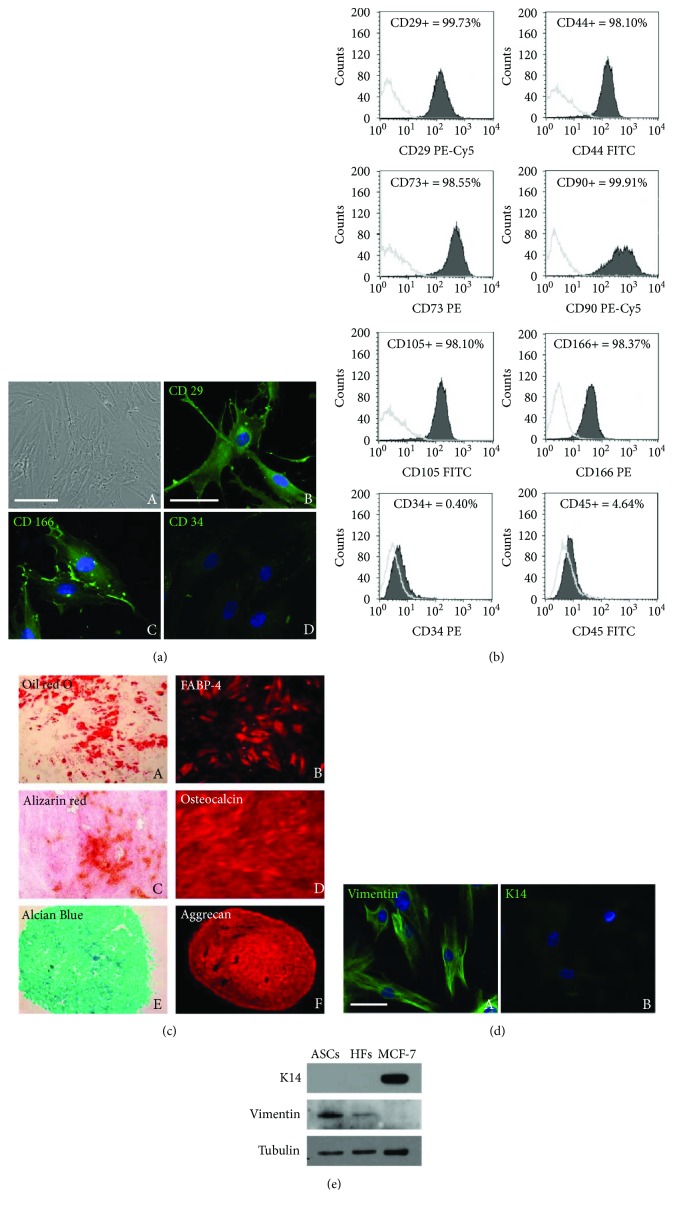
Phenotypical characterization of ASCs. (a) Representative phase-contrast photomicrograph of ASCs, showing homogeneous, spindle-shaped morphology (A) and immunofluorescence analysis showing positive staining for the mesenchymal antigens CD29 (B) and CD166 (C) and negative staining for the hematopoietic CD34 antigen (D). Nuclei (blue) were visualized with 4′,6-diamidino-2-phenylindole (DAPI). Scale bars: 100 *μ*m. (b) Flow cytometric analysis of ASCs. Cells were stained with monoclonal antibodies directed against CD29, C44, CD90, CD166, CD73, CD105, CD34, and CD45. Dark grey areas represent patterns obtained with antibodies against the indicated markers, whereas light grey lines represent the isotype-matched monoclonal antibody that served as a control. Each panel reported the percentage of positive cells for the corresponding marker. (c) Multilineage differentiation of ASCs. Adipogenic differentiation was assessed by positive staining with oil red O (A) and by positive immunofluorescence analysis for the adipocyte-specific fatty acid binding protein 4 (FABP4) (B). Osteogenic differentiation was demonstrated by positive staining for alizarin red (C) and positive immunofluorescence reactivity to osteocalcin (D). Chondrogenic differentiation was assessed by positive staining for Alcian Blue (E) and by positive immunofluorescence with anti-aggrecan antibodies (F). (d) Immunofluorescence analysis of ASCs showing positive staining for the mesenchymal marker vimentin (A) and negative staining for the epithelial marker cytokeratin 14 (K14) (B). Scale bars: 100 *μ*m. (e) Expression of vimentin and K14 assessed by Western blot analysis on ASC whole cell lysates. HFs and MCF-7 cells were used as controls for mesenchymal and epithelial lineage, respectively. Western blot with anti-tubulin antibody served as loading control. The images are representative of at least three independent experiments.

First, we obtained primary cultures of ASCs from adipose tissue. Cultured ASCs showed the typical spindle-shape phenotype of mesenchymal stem cells, as observed by phase contrast microscopy (Figure 1(a), A). We then analyzed the specific pattern of cell surface markers in our ASC cultures by immunofluorescence (Figure 1(a), B–D) and flow cytometry (Figure 1(b)), in order to exclude the presence of contaminating elements such as hematopoietic stem cells. ASC characterization was performed using the expression of cell-specific surface markers, according to the criteria reported elsewhere [1]. In particular, CD29, CD44, CD73, CD90, CD105, and CD166 were considered as typical mesenchymal stemness markers, while CD34 and CD45 were assumed as expressed by hematopoietic stem cells. Immunofluorescence analysis confirmed that our cells expressed the CD29 and CD166 (Figure 1(a), B and C) and were negative for CD34 (Figure 1(a), D). Moreover, flow cytometry on ASCs confirmed the expression of CD29, CD44, CD73, CD90, CD105, and CD166, whereas no expression of CD34 or CD45 was detected (Figure 1(b)). We also assessed the capability of ASCs to differentiate into various cell types. The adipogenic, osteogenic, and chondrogenic differentiation was induced for 21 days and assessed as previously described [27]. In particular, cells subjected to adipogenic differentiation showed evident intracellular lipid accumulation, detected by oil red O staining (Figure 1(c), A) and the expression of the lineage-specific FABP4 protein (Figure 1(c), B). ASCs induced towards osteogenic differentiation showed deposition of de novo bone matrix, as assessed by alizarin red S staining (Figure 1(c), C), and the expression of the bone-specific protein osteocalcin (Figure 1(c), D). Finally, cell pellets induced towards chondrogenic differentiation showed secretion of sulfated glycosaminoglycans, as indicated by positive stain for Alcian Blue (Figure 1(c), E) and the expression of the large proteoglycan aggrecan (Figure 1(c), F). ASCs were further characterized for the expression of mesenchymal and epithelial markers (vimentin and cytokeratin 14, resp.) by both immunofluorescence and Western blot analysis, to confirm the absence of epithelial contaminants. Consistently with their mesenchymal origin, ASCs were positive for vimentin (Figure 1(d), A; Figure 1(e)), while no expression of cytokeratin 14 (K14) was detected in our cultures (Figure 1(d), B; Figure 1(e)). Thus, we were able to obtain primary cultures of ASCs devoid of contaminating elements, such as hematopoietic stem cells or epithelial cells.

### 3.2. KGF Expression during ASC Adipogenesis

**Figure 2:**
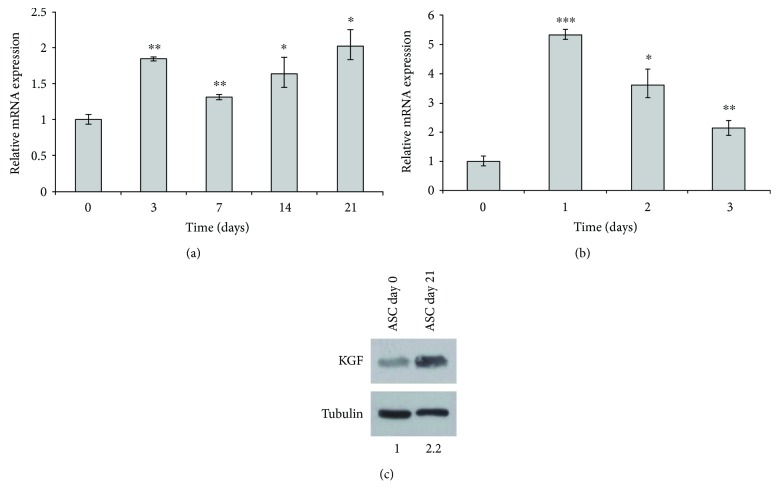
qRT-PCR analysis of KGF mRNA expression in ASCs at different times of adipogenic differentiation. (a) Relative KGF mRNA levels at days 3, 7, 14, and 21 of adipogenic differentiation are shown as fold value of the level of KGF mRNA in undifferentiated cells (day 0). (b) Relative KGF mRNA levels at days 1, 2, and 3 of adipogenic differentiation are shown as fold value of the level of KGF mRNA in undifferentiated cells (day 0). Each experiment was performed in triplicate, and mRNA levels were normalized to PPIA mRNA expression. Error bars represent standard deviations. ^∗^ *P* < 0.05; ^∗∗^ *P* < 0.005; and ^∗∗∗^ *P* < 0.0005. (c) KGF protein expression was assessed by Western blot analysis with anti-FGF7 antibody on ASC whole cell lysates at days 0 and 21 of adipogenic differentiation. Tubulin served as loading control.

To assess the potential involvement of KGF in adipogenesis, we induced our cells through adipogenic differentiation by means of a medium containing specific supplements (ADM) and assessed the transcript levels of KGF in ASCs on several differentiation times (0, 3, 7, 14, and 21 days) by qRT-PCR (Figure 2(a)). We found that KGF expression fluctuated during ASC differentiation, but it was always higher than that in undifferentiated cells (day 0). In particular, we observed maximal upregulation in early and late differentiation (day 3, 1.9-fold increase, *P* < 0.005, and day 21, 2.0-fold increase, *P* < 0.05, resp.). To better characterize the involvement of KGF in the early steps of adipogenesis, we assessed its expression during the first 3 days of differentiation (Figure 2(b)), and we observed a peak of KGF expression at day 1 of adipogenic induction (5.3-fold increase, *P* < 0.0005), thus suggesting a potential role of KGF upregulation in early steps of adipogenesis. We also performed WB analysis on ASCs at day 0 and at day 21 of adipogenic differentiation, to assess KGF protein expression (Figure 2(c)). Even at protein level, differentiated ASCs showed an increase in KGF expression (2.2-fold), thus confirming the data obtained at mRNA level by real-time PCR.

### 3.3. KGF Effect on ASC Proliferation

**Figure 3:**
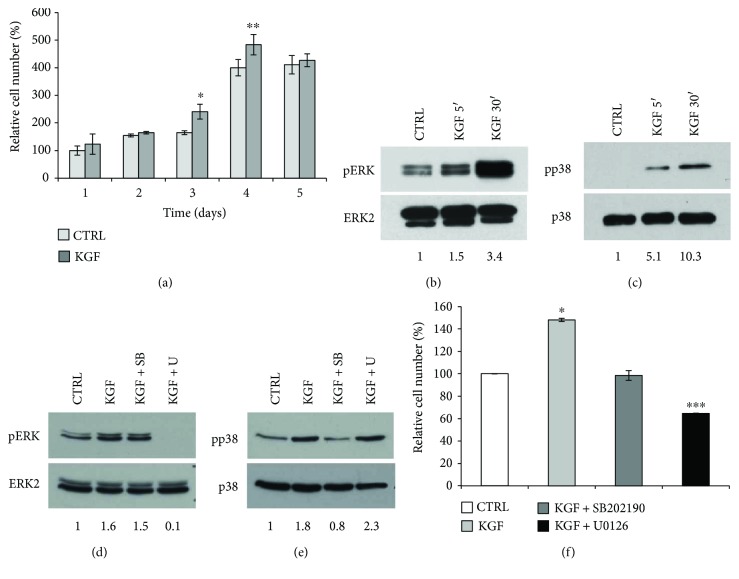
Evaluation of KGF effect on ASC proliferation. (a) Proliferation ability of ASCs treated or not with 20 ng/ml KGF for 1–5 days was determined by MTT assay. Error bars represent standard deviations from three independent experiments. ^∗^ *P* < 0.05 and ^∗∗^ *P* < 0.005. (b) Phosphorylation of ERK was assessed by Western blot analysis with a phospho-specific ERK monoclonal antibody (pERK) on ASC whole cell lysates, treated or not with 20 ng/ml KGF for 5 and 30 min. Levels of total ERK were assessed by blotting with an ERK2-specific antibody and served as loading control. (c) Phosphorylation of p38 was assessed by Western blot analysis with a phospho-specific p38 monoclonal antibody (pp38) on ASC whole cell lysates, treated or not with 20 ng/ml KGF for 5 and 30 min. Levels of total p38 were assessed by blotting with a p38-specific antibody and served as loading control. (d) Phosphorylation of ERK was assessed by Western blot analysis with a phospho-specific ERK monoclonal antibody (pERK) on ASC whole cell lysates, treated or not with 20 ng/ml KGF for 5 min, in the presence or not of the p38 inhibitor SB202190 or of the ERK inhibitor U0126. Levels of total ERK were assessed by blotting with an ERK2-specific antibody and served as loading control. (e) Phosphorylation of p38 was assessed by Western blot analysis with a phospho-specific p38 monoclonal antibody (pp38) on ASC whole cell lysates, treated or not with 20 ng/ml KGF for 30 min, in the presence or not of the p38 inhibitor SB202190 or of the ERK inhibitor U0126. Levels of total p38 were assessed by blotting with a p38-specific antibody and served as loading control. (b–e) The intensity of the bands was evaluated by densitometric analysis; the values from at least three independent experiments were normalized and reported as fold increase with respect to the untreated sample. (f) Proliferation ability of ASCs treated or not with 20 ng/ml KGF in the presence or not of the p38 inhibitor SB202190 or of the ERK inhibitor U0126 for 3 days was determined by MTT assay. Error bars represent standard deviations from three independent experiments. ^∗^ *P* < 0.05 and ^∗∗∗^ *P* < 0.0005.

To determine whether KGF could also induce ASC proliferation, cells were serum starved and then treated with KGF for 1, 2, 3, 4, or 5 days, and proliferation was assessed through MTT assay (Figure 3(a)). No increase in cell number was observed during the first 2 days, which is compatible with the long population doubling time of these cells [27]. At 3 and 4 days of exposure to KGF, we observed a significant induction of cell proliferation (46%, *P* < 0.005, and 21%, *P* < 0.05, resp.), which is not maintained until day 5, probably due to KGF degradation.

To assess the mechanism of KGF-mediated proliferation in ASC cultures, we analyzed the activation of the extracellular signal-regulated kinase (ERK) pathway and the p38MAPK pathway. We found a consistent activation of ERK1/2 after 5 and 30 min of treatment with KGF (1.5-fold and 3.4-fold, resp.; Figure 3(b)). KGF treatment was also able to strongly phosphorylate the p38 protein at the same time intervals (5.1-fold and 10.3-fold, resp.; Figure 3(c)), thus suggesting that both these pathways could be involved in KGF-mediated ASC proliferation.

To better address the involvement of ERK and p38MAPK pathways in KGF-induced ASC proliferation, we decided to assess ASC proliferation in the presence of selective inhibitors for these two pathways. First, the efficacy of the ERK inhibitor U0126 and of the p38MAPK inhibitor SB202190 was confirmed by Western blot analysis of ERK and p38 phosphorylation levels after treatment with KGF in the presence or not of each inhibitor (Figures 3(d) and 3(e)). As expected, after 5 min of treatment with KGF, we found activation of ERK1/2 (1.6-fold, *P* < 0.0005; Figure 3(d) and of p38 protein (1.8-fold, *P* < 0.05; Figure 3(e)). Cotreatment with SB202190 selectively inhibited p38 phosphorylation (0.8-fold; Figure 3(e)), without affecting ERK phosphorylation (1.5-fold, *P* < 0.05; Figure 3(d)). Conversely, treatment with U0126 strongly inhibited KGF-mediated ERK phosphorylation (0.1-fold, *P* < 0.0005; Figure 3(d)) without affecting activation of p38 protein (2.3-fold, *P* < 0.05; Figure 3(e)). Then, we assessed ASC proliferation. Briefly, cells were serum starved and then treated for 3 days with KGF alone or in combination with the ERK inhibitor U0126 or with the p38MAPK inhibitor SB202190, and proliferation was assessed through MTT assay (Figure 3(f)). In KGF-treated cells, we observed a significant induction of cell proliferation (48%, *P* < 0.05), as expected. Interestingly, no increase in cell number was achieved when KGF treatment was performed in the presence of the p38MAPK inhibitor SB202190, and even a reduction in cell number was evident when ASCs were treated with KGF in combination with the ERK inhibitor U0126 (−35.4%, *P* < 0.0005), thus confirming the involvement of both these pathways in KGF-mediated ASC proliferation.

### 3.4. KGF Effect on ASC Differentiation

**Figure 4:**
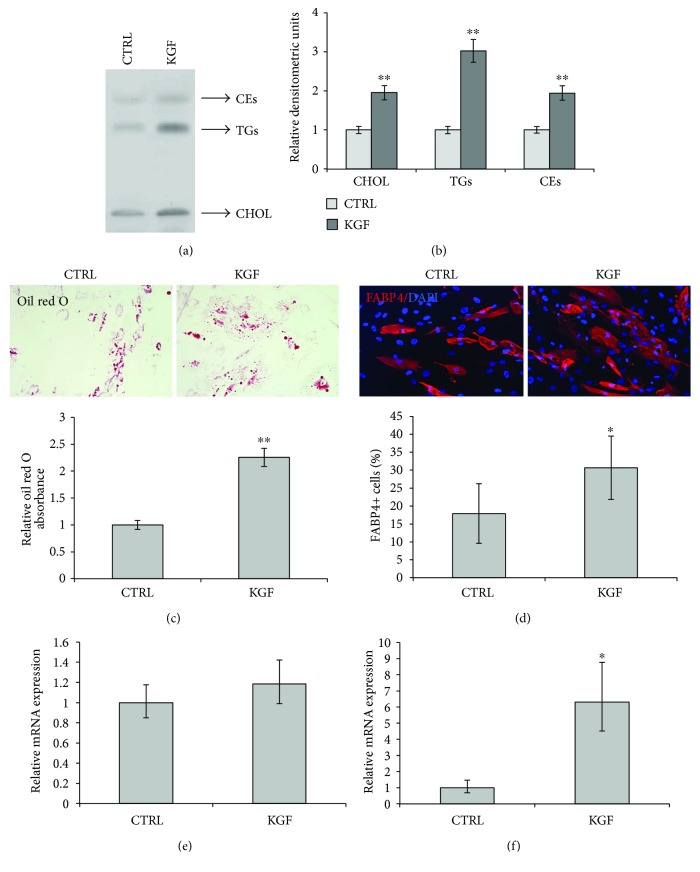
Evaluation of KGF effect on ASC adipogenic differentiation. (a) HPTLC analysis of the neutral-lipid cholesterol (CHOL), triglycerides (TGs), and cholesterol esters (CEs) in ASCs treated or not with 20 ng/ml KGF for 24 h. (b) The intensity of the bands was evaluated by densitometric analysis, normalized and reported in a graph as relative expression with respect to untreated cells. Error bars represent standard deviations (^∗∗^ *P* < 0.005). (c) Representative images of ASCs cultured in adipogenic medium with or without KGF for 21 days and subjected to lipid staining with oil red O. Stained cells were then solubilized using isopropanol, and the extent of adipocyte differentiation was quantitated by determining the amount of extracted dye, as measured by the optimal absorbance at 490 nM, reported in the graph as relative expression with respect to untreated cells (^∗∗^ *P* < 0.005). (d) Immunofluorescence analysis of the adipocyte-specific fatty acid binding protein 4 (FABP4, red) in ASCs at day 21 of adipogenic differentiation, treated or not with KGF during differentiation. Nuclei (blue) were visualized with 4′,6-diamidino-2-phenylindole (DAPI). The percentage of FABP4-positive cells was determined by counting the number of FABP4-positive cells versus total number of cells in ten different areas randomly taken from three independent experiments. Error bars represent standard deviations (^∗^ *P* < 0.05). (e) Relative PPAR*γ* mRNA levels at day 21 of adipogenic differentiation in ASCs treated with KGF are shown as fold value of the level of PPARγ mRNA in untreated cells. Error bars represent standard deviations. (f) Relative FABP4 mRNA levels at day 21 of adipogenic differentiation in ASCs treated with KGF are shown as fold value of the level of FABP4 mRNA in untreated cells. Error bars represent standard deviations (^∗^ *P* < 0.05).

We assessed the effect of KGF treatment on ASC neutral lipid content. ASCs were cultured with and without KGF for 24 h, and then lipids were extracted and analyzed by high-performance thin-layer chromatography. As reported in Figures 4(a) and 4(b), KGF treatment significantly increased the amount of free cholesterol (CHOL), triglycerides (TGs), and cholesterol esters (CEs) (2.0-fold, 3.0-fold, and 1.9-fold, resp., *P* < 0.005).

We then analyzed the effect of prolonged KGF treatment on ASCs induced to differentiate towards the adipogenic lineage by a standard protocol. At day 21 of differentiation, cells treated or not with KGF were stained with oil red O. The quantitation of solubilized oil red O dye showed that the amount of lipid droplets was higher in KGF-treated cells with respect to control cells (CTRL) (2.3-fold, *P* < 0.005; Figure 4(c)). Cells were also subjected to immunofluorescence analysis with the adipocyte marker FABP4 (Figure 4(d)). KGF treatment determined an increase of the percentage of FABP4-positive cells (30.6% versus 17.9% of untreated cells (CTRL), *P* < 0.05). Since KGF-treated ASCs demonstrated enhanced differentiation than the control cells, we further investigated whether KGF could regulate the expression of specific genes involved in adipogenesis by qRT-PCR. At 21 days of adipogenic differentiation, the expression levels of PPAR*γ* in KGF-treated cells showed only a slight increase with respect to untreated cells (CTRL) (1.2-fold; Figure 4(e)), while the levels of FABP4 were significantly higher in KGF-treated cells (6.3-fold, *P* < 0.05; Figure 4(f)), thus strengthening the data obtained with immunofluorescence analysis.

**Figure 5:**
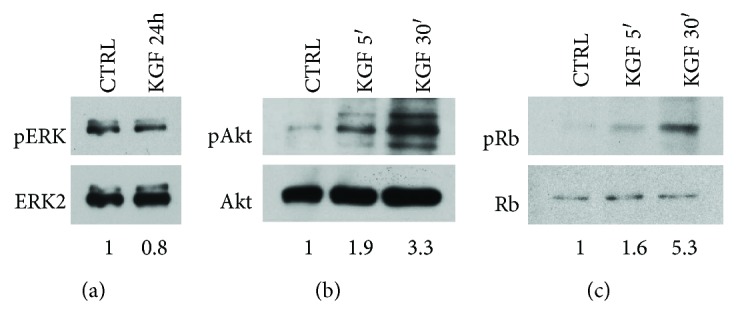
Evaluation of differentiation pathway activation by KGF. (a) Sustained phosphorylation of ERK was assessed by Western blot analysis with a phospho-specific ERK monoclonal antibody (pERK) on ASC whole cell lysates, treated or not with 20 ng/ml KGF for 24 h. Levels of total ERK were assessed by blotting with an ERK2-specific antibody and served as loading control. (b) Phosphorylation of Akt was assessed by Western blot analysis with a phospho-specific Akt monoclonal antibody (pAkt) on ASC whole cell lysates, treated or not with 20 ng/ml KGF for 5 and 30 min. Levels of total Akt were assessed by blotting with an Akt-specific antibody and served as loading control. (c) Phosphorylation of Rb was assessed by Western blot analysis with a phospho-specific Rb monoclonal antibody (pRb) on ASC whole cell lysates, treated or not with 20 ng/ml KGF for 5 and 30 min. Levels of total Rb were assessed by blotting with a Rb-specific antibody and served as loading control. The images are representative of at least three independent experiments.

In order to clarify the mechanism by which KGF affects cell differentiation, we first assessed its effect on sustained ERK activation, since the activation of differentiation program requires the switch off of ERK signaling. We demonstrated that in ASCs, KGF promotes only transient ERK activation, with no detection of ERK phosphorylation after 24 h of KGF treatment (Figure 5(a)).

Then, we decided to examine the effect of KGF on pathways involved in cell differentiation, such as those of PI3K/Akt and the retinoblastoma protein (Rb). As reported in Figure 5(b), KGF was able to induce Akt phosphorylation, at both 5 and 30 min (1.9-fold and 3.3-fold, resp.). We also observed in ASCs a significant phosphorylation of Rb protein induced by KGF after 5 min of treatment, and more consistently after 30 min (1.6-fold and 5.3-fold, resp.; Figure 5(c)).

Taken together, these data demonstrate that KGF is involved in ASC proliferation and adipogenesis, by promotion of multiple pathways stimulation. Furthermore, such evidence underscores the presence in these cells of a membrane receptor that is able to mediate KGF downstream signaling.

### 3.5. Expression and Functionality of FGFR2-IIIb in ASCs

**Figure 6:**
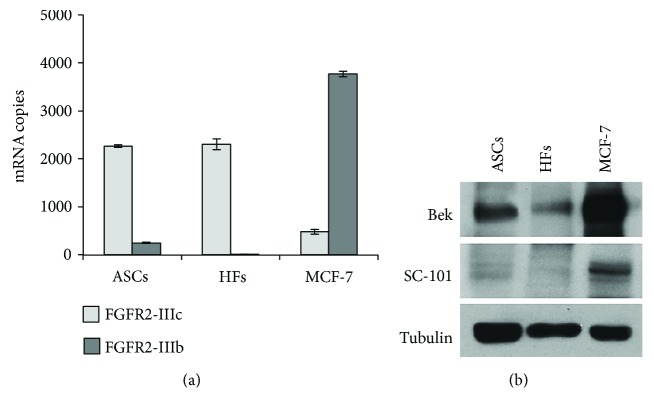
FGFR2-IIIb and FGFR2-IIIc expression in ASCs. (a) FGFR2-IIIb and FGFR2-IIIc gene expression in ASCs were measured by absolute quantitation real-time PCR. MCF-7 cells were used as a positive control for FGFR2-IIIb and HFs as positive control for FGFR2-IIIc. (b) Western blot analysis of FGFR2-IIIb protein levels in ASCs, HFs, and MCF-7 cells. FGFR2-IIIb protein expression was evaluated by blotting with an anti-Bek antibody or with the homemade FGFR2-IIIb-specific SC-101 mAb. Western blot with anti-tubulin antibody was used as loading control. The images are representative of at least three independent experiments.

KGF is known to act by binding to the FGFR2-IIIb isoform (also known as KGFR), expressed by cells of epithelial origin, whereas the alternatively spliced FGFR2-IIIc isoform is generally expressed in mesenchymal cells. We decided to investigate the expression of both FGFR2 isoforms in ASCs, through absolute quantification of transcripts by qRT-PCR. Briefly, using a series of diluted plasmid DNA as templates, we obtained a standard curve for FGFR2-IIIb and for FGFR2-IIIc, from which we obtained the exact copy number of both isoforms in ASCs, as well as in human fibroblasts (HFs), used as control for the mesenchymal lineage, and in the human breast cancer cell line MCF-7, used as control for the epithelial lineage (Figure 6(a)). As expected, we observed a high expression of the mesenchymal isoform FGFR2-IIIc in both ASCs and HFs (about 2263 and 2302 copies/25 ng total RNA, resp.), and a strong expression of FGFR2-IIIb in the epithelial cell line MCF-7 (3768 copies), which also express a small amount of FGFR2-IIIc (482 copies), due to their neoplastic nature, as previously observed [32, 33]. HFs did not express a significant amount of FGFR2-IIIb (16 copies), but, surprisingly, we detected a certain amount of this isoform in ASCs (252 copies).

Therefore, to assess if the presence of FGFR2-IIIb transcript in these cells would correspond to a significant amount of protein, we performed Western blot analysis on the same cellular models. Western blot with a commercial antibody directed against FGFR2 (Bek), which does not discriminate the two isoforms FGFR2-IIIb and FGFR2-IIIc, showed a consistent expression of FGFR2 protein in all the three type of cells (Figure 6(b)). Then, we assessed FGFR2-IIIb expression by using a homemade antibody [31], which is able to specifically recognize this isoform. As shown in Figure 6(b), Western blot analysis for FGFR2-IIIb confirmed a consistent expression in MCF-7 cells and an almost undetectable expression in HFs. However, we detected a faint but not negligible expression of FGFR2-IIIb in ASCs, thus confirming the data obtained by qRT-PCR.

These results demonstrate for the first time that ASCs, despite their mesenchymal nature, are able to synthesize and express a low amount of FGFR2-IIIb mRNA and protein.

**Figure 7:**
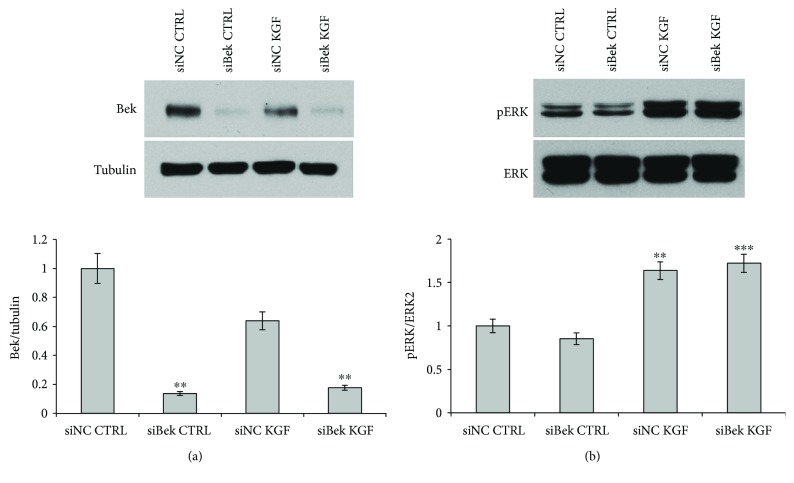
Effect of FGFR2 silencing on KGF-mediated phosphorylation of ERK. (a) ASCs transfected with FGFR2-specific siRNA (siBek) or nonspecific control siRNA (siNC), treated or not with 20 ng/ml KGF for 5 min at 37°C, were lysed, and FGFR2 expression was analyzed by immunoblotting with anti-Bek antibodies. siBek induced a marked reduction in FGFR2 expression in both untreated and KGF-treated cells. Western blot with anti-tubulin antibodies was used as loading control. (b) The same lysates were analyzed by immunoblotting with anti-phospho-ERK antibody. Transfection with siBek did not affect ERK phosphorylation levels in KGF-treated cells. The levels of total ERK were assessed by Western blot with anti-ERK1/2 antibodies. The intensity of the bands was evaluated by densitometric analysis; the values from a representative experiment were normalized, expressed as fold increase with respect to the untreated siNC sample and reported as a graph. ^∗∗^ *P* < 0.005 and ^∗∗∗^ *P* < 0.0005.

So, we wondered if this low amount of FGFR2-IIIb in ASCs would be sufficient to support KGF-mediated pathway activation in these cells. For this purpose, we achieved the downmodulation of FGFR2 gene expression in ASCs by transfection with an FGFR2-specific small interfering RNA (siBek). A nonspecific siRNA was used as negative control (siNC). First, we analyzed the efficiency of FGFR2 silencing by Western blot with Bek polyclonal antibodies in ASCs treated or not with KGF for 5 min at 37°C. Tubulin was used to assess the equal loading (Figure 7(a)). SiBek specifically reduced FGFR2 expression by more than 80%, in both untreated (CTRL) and KGF-treated cells (0.1- and 0.2-fold increase, resp., *P* < 0.005), with respect to treatment of cells with control siRNA (siNC) (Figure 7(a)). It should be noticed that KGF treatment induced a decrease in FGFR2 protein levels, probably due to receptor endocytosis and lysosomal degradation (see Figure S1 for time course experiments). Then, we investigated the effect of FGFR2 downregulation on ERK activation induced by KGF. As shown in Figure 7(b), transfection of ASCs with FGFR2 siRNA did not lead to a significant reduction in ERK phosphorylation induced by KGF (1.7-fold for siBek versus 1.6-fold for siNC), thus suggesting that KGF activity in these cells is not carried out through FGFR2-IIIb, but is presumably mediated by a previously hypothesized unknown receptor.

### 3.6. Identification of a Potential Alternative Receptor for KGF in ASCs

In order to find another receptor that might be responsible for KGF pathway activation in ASCs, we analyzed the KGF Entrez Gene database [34]. The first interactant gene reported in the “Interactions” section was Neuropilin 1 (NRP1), a known coreceptor for other growth factors, such as VEGF-A or PDGF. Such interaction has been demonstrated by West et al. [35].

**Figure 8:**
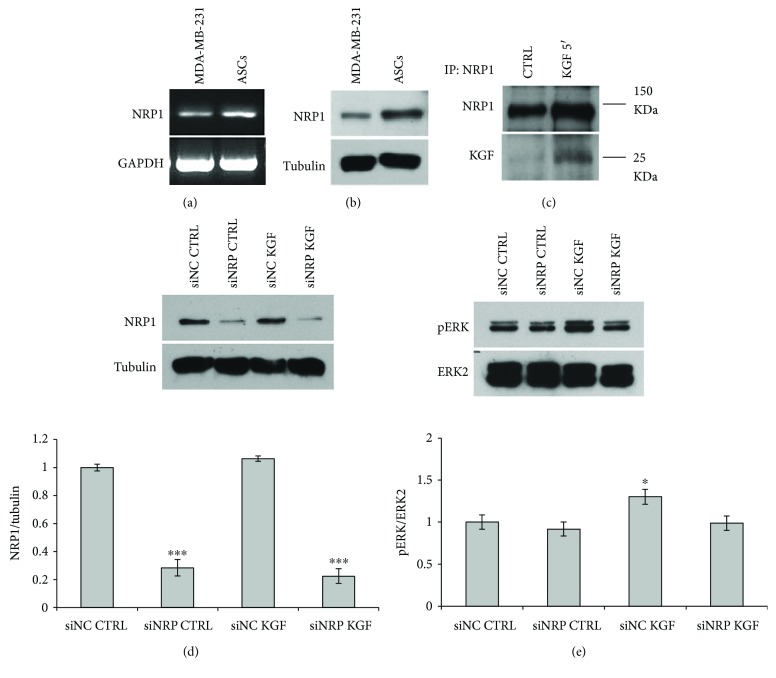
Effect of NRP1 silencing on KGF-mediated phosphorylation of ERK. Expression of NRP1 assessed by PCR (a) and Western blot analysis (b) on ASC whole cell lysates. MDA-MB-231 cells were used as positive control for NRP1 expression. GAPDH mRNA expression and blotting with anti-tubulin antibody served as loading control for PCR and Western blot analysis, respectively. The images are representative of at least three independent experiments. (c) Coimmunoprecipitation assay was performed to study in vivo interaction between KGF and NRP1 proteins. ASCs, untreated or treated with 20 ng/ml KGF for 5 min, were immunoprecipitated with anti-NRP1 antibody and blotted with anti-FGF7 antibody. Western blot with anti-NRP1 antibody was used as loading control. (d) ASCs transfected with NRP1-specific siRNA (siNRP) or nonspecific control siRNA (siNC), treated or not with 20 ng/ml KGF for 5 min at 37°C, were lysed, and NRP1 expression was analyzed by immunoblotting with anti-NRP1 antibodies. siNRP induced a marked reduction in NRP1 expression in both untreated and KGF-treated cells. Western blot with anti-tubulin antibodies was used as loading control. (e) The same lysates were analyzed by immunoblotting with anti-phospho-ERK antibody. Transfection with siNRP significantly inhibits ERK phosphorylation induced by KGF treatment. The levels of total ERK were assessed by Western blot with anti-ERK1/2 antibodies. The intensity of the bands was evaluated by densitometric analysis; the values from a representative experiment were normalized, expressed as fold increase with respect to the untreated siNC sample and reported as a graph. ^∗^ *P* < 0.05 and ^∗∗∗^ *P* < 0.0005.

We first assessed that NRP1 is expressed in ASCs, both at the RNA and protein level (Figures 8(a) and 8(b), resp.). MDA-MB-231 cells were used as positive control for Neuropilin expression. Then, to determine whether the NRP1 receptor was in a molecular complex with the KGF protein, the ability of KGF to coprecipitate with the NRP1 receptor was assessed in ASCs treated or not with 20 ng/ml KGF for 5 min. Protein lysates were immunoprecipitated with anti-NRP1 antibody, and the immunoprecipitates were probed for the presence of KGF or of NRP1 receptor by Western blotting with the respective antibodies. KGF protein was detected in NRP1 immunoprecipitates (Figure 8(c)) after KGF treatment, thus indicating that KGF and NRP1 are able to interact in ASCs. Such data prompted us to investigate the possible involvement of NRP1 in ERK activation induced by KGF. For this purpose, we downregulated the expression of NRP1 in ASCs by means of a specific siRNA (siNRP). The efficiency of NRP1 silencing in ASCs treated or not with KGF for 5 min was assessed by Western blot with an anti-NRP1 antibody. Tubulin was used to assess the equal loading (Figure 8(d)). NRP1 silencing was achieved with an efficiency of about 70–80%, in both untreated and KGF-treated cells (0.3-fold and 0.2-fold, resp., *P* < 0.0005), with respect to treatment of cells with control siRNA (siNC) (Figure 8(d)). In this case, we did not observe a significant decrease of NRP1 protein levels after treatment with KGF for 5′, as previously shown for FGFR2 (Figure 7(a)). To better address this point, since such a phenomenon would be expected if NRP1 functions as a receptor for KGF in ASCs, we performed time course experiments to assess the protein levels of FGFR2 and NRP1 after KGF treatment. Cells were treated with KGF for 5′, 15′, 30′, 1 h, 2 h, and 3 h and then subjected to WB analysis for FGFR2 and NRP1 (Figure S1). We observed, as expected, a significant decrease of FGFR2 levels just after 5′ of treatment (*P* < 0.0005), in accordance to what was previously shown in Figure 7(a), then FGFR2 levels remains significantly lower than those of untreated cells until 2 h of treatment (*P* < 0.0005). Concerning NRP1, we observed, although to a lesser extent and starting at longer time of KGF exposure, a significant decrease of its expression after 15′ and 30′ of KGF treatment (*P* < 0.0005 and *P* < 0.005, resp.). Therefore, the data obtained from time course experiments are not in conflict with a potential role of NRP1 as KGF receptor.

**Figure 9:**
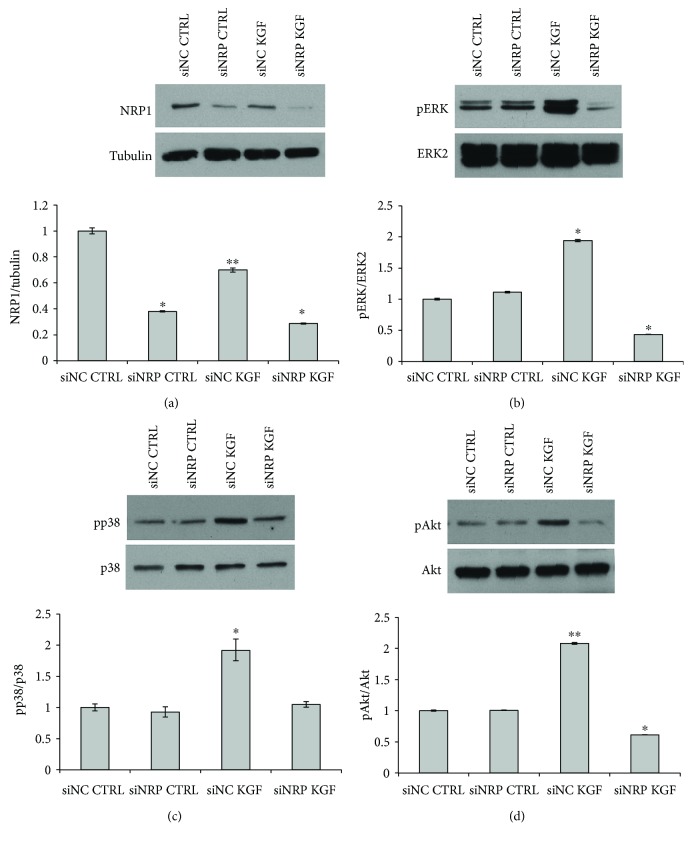
Effect of NRP1 silencing on KGF-mediated phosphorylation of ERK, p38, and Akt. (a) ASCs transfected with NRP1-specific siRNA (siNRP) or nonspecific control siRNA (siNC), treated or not with 20 ng/ml KGF for 30 min at 37°C, were lysed, and NRP1 expression was analyzed by immunoblotting with anti-NRP1 antibodies. siNRP induced a marked reduction in NRP1 expression in both untreated and KGF-treated cells. Western blot with anti-tubulin antibodies was used as loading control. (b) The same lysates were analyzed by immunoblotting with anti-phospho-ERK antibody. Transfection with siNRP significantly inhibits ERK phosphorylation induced by KGF treatment. The levels of total ERK were assessed by Western blot with anti-ERK1/2 antibodies. (c) Phosphorylation of p38 was assessed by Western blot analysis with a phospho-specific p38 monoclonal antibody (pp38). Levels of total p38 were assessed by blotting with a p38-specific antibody and served as loading control. (d) Phosphorylation of Akt was assessed by Western blot analysis with a phospho-specific Akt monoclonal antibody (pAkt). Levels of total Akt were assessed by blotting with an Akt-specific antibody and served as loading control.The intensity of the bands was evaluated by densitometric analysis; the values from a representative experiment were normalized, expressed as fold increase with respect to the untreated siNC sample and reported as a graph. ^∗^ *P* < 0.05 and ^∗∗^ *P* < 0.005.

Then, we analyzed ERK activation induced by KGF in NRP1-silenced cells. As shown in Figure 8(e), NRP1 downregulation significantly inhibited KGF-dependent ERK phosphorylation (1.0-fold for siNRP versus 1.3-fold for siNC). To further assess NRP1 role in KGF-mediated signaling pathways, we analyzed phosphorylation of ERK, p38MAPK, and Akt in NRP1-silenced cells at 30 min of KGF treatment (since previous experiments indicated a stronger pathway activation at this time point, see Figures 3 and 5). As reported in Figure 9, we first assessed the efficiency of NRP1 silencing in ASCs treated or not with KGF for 30 min by Western blot with an anti-NRP1 antibody. Tubulin was used to assess the equal loading (Figure 9a). NRP1 silencing was achieved in both untreated and KGF-treated cells (0.4-fold and 0.3-fold, resp., *P* < 0.05), with respect to treatment of cells with control siRNA (siNC). After NRP1 silencing, we observed a significant reduction in KGF-induced activation of ERK (0.4-fold for siNRP versus 1.9-fold for siNC), p38 (1.0-fold for siNRP versus 1.9-fold for siNC), and Akt (0.6-fold for siNRP versus 2.1-fold for siNC).

In conclusion, NRP1 seems to be directly responsible for KGF-dependent pathway activation in ASCs, thus suggesting its role as an alternative receptor for this growth factor in these cells.

### 3.7. FGFR2-IIIb and NRP1 Expression during ASC Adipogenesis

**Figure 10:**
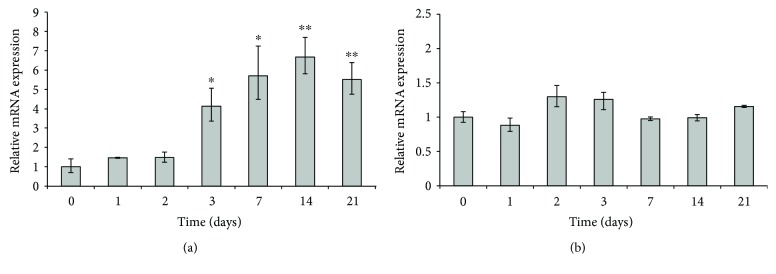
qRT-PCR analysis of FGFR2-IIIb (a) and NRP1 (b) mRNA expression in ASCs at different times of adipogenic differentiation. Relative mRNA levels at days 1, 2, 3, 7, 14, and 21 of adipogenic differentiation are shown as fold value of the level of mRNA in undifferentiated cells (day 0). Each experiment was performed in triplicate, and mRNA levels were normalized to PPIA mRNA expression. Error bars represent standard deviations. ^∗^ *P* < 0.05 and ^∗∗^ *P* < 0.005.

To assess the role of FGFR2-IIIb and NRP1 in adipogenesis, we induced our cells through adipogenic differentiation by means of ADM medium and assessed the transcript levels of both receptors in ASCs on several differentiation times (0, 1, 2, 3, 7, 14, and 21 days) by qRT-PCR (Figure 10). We found that FGFR2-IIIb expression (Figure 10(a)) started to increase at day 3 of ASC differentiation. In particular, we observed maximal upregulation in middle-late differentiation (day 7, 5.7-fold increase, *P* < 0.05, and day 14, 6.7-fold increase, *P* < 0.005, resp.). As for NRP1 (Figure 10(b)), its expression is holding steady during all the differentiation process, with a slight increase peaking at days 2 and 3 of adipogenic induction (1.3-fold increase), thus suggesting a potential role of NRP1 in the response of ASCs to endogenous KGF upregulation in early steps of adipogenesis (see Figure 2(b)).

## 4. Discussion

The present work provides a novel insight into the role of KGF in human adipogenesis of ASCs. It is well known that ASCs, as well as bone marrow stem cells (BMSCs), release soluble paracrine factors, including KGF, which are crucial for the usefulness of these cells in therapeutic strategies to restore structure and function of epithelial tissues. However, at present, there is no evidence of a direct effect of KGF on BMSCs. Interestingly, recent evidence indicates a role of KGF in the differentiation of human umbilical cord-derived mesenchymal stem cells (hUC-MSCs) into sweat gland-like cells (SGCs), with potential implications in the field of regeneration of destroyed sweat glands and injured skin [36]. Moreover, a role of KGF in promoting proliferation pathways on murine preadipocytes [21], and in inducing the expression of key regulators of adipogenesis in alveolar type II cells [22], has been previously reported. However, the role of KGF in human ASC proliferation and adipogenesis still needs to be elucidated. In contrast to what observed in murine preadipocytes [21], we demonstrated a significant change in levels of KGF mRNA expression throughout ASC adipogenic differentiation, with a peak at day 1 of differentiation. This observation suggests that the upregulation of KGF expression might be required for the proper induction of ASC differentiation and prompted us to further investigate the role of KGF in adipogenesis.

Since an effect of KGF on preadipocytes proliferation has been previously demonstrated in a murine model [21], we determined whether KGF could also induce ASC proliferation. We decided to analyze the activation of the extracellular signal-regulated kinase (ERK) pathway and the p38MAPK pathway, since they are both indicated as required not only for mesenchymal cell proliferation but also for the mitotic clonal expansion that represent the first step of adipogenesis [37, 38]. Based on our results, we suggest that KGF might promote the proliferation of ASCs and the mitotic clonal expansion to initiate adipogenesis through activation of ERK and p38MAPK pathways. Moreover, in contrast with what observed by Zhang et al. [21], we demonstrated that in human ASCs, proliferation and differentiation are not mutually exclusive, since KGF is also able to promote adipogenic differentiation. This double role in adipogenesis has been previously demonstrated also for other growth factors and receptors, such as members of the Notch family and of the FGF family [5, 12, 39].

However, the role of ERK and p38MAPK pathways in adipogenesis is still controversial. In the case of ERK, its activation seems to be necessary for the initial proliferative step, but the pathway needs to be shut off to allow proper differentiation [38]. Our data are consistent with this observation, since we demonstrated that KGF induces only transient ERK activation, in which phosphorylation is no longer detectable after 24 h of KGF treatment. Concerning p38MAPK pathway, opposing roles in adipocyte differentiation have been described, but Aouadi et al. demonstrated a positive role of this pathway in human adipogenesis through regulation of adipogenic transcription factors [40]. Therefore, our evidence of p38MAPK activation induced by KGF is not in contrast with a role of this growth factor in ASC differentiation. Moreover, this role is sustained by our observation of KGF-mediated stimulation of PI3K/Akt and Rb pathways, which are known to be important for adipogenic differentiation [41, 42]. In particular, it has been reported that Akt plays a role in adipogenesis in both murine 3T3-L1 preadipocyte [43] and human ASCs [44]. Concerning the retinoblastoma protein Rb, its role in adipogenesis is complex. Phosphorylation-dependent inactivation of Rb permits progression through mitotic clonal expansion, by releasing the members of the E2F family of transcription factors [19]. However, Rb has also an important role in cell cycle exit, as well as its interaction with members of the C/EBP family of transcription factors. In particular, Rb has been demonstrated to stimulate adipogenesis though activation of C/EBP*α* [45]. Early alterations in Rb phosphorylation may be important for the antiadipogenic effect of macrophage-secreted factors on 3T3-L1 adipocyte differentiation [46]. Other studies indicate that pRb acts as a molecular switch determining white versus brown adipogenesis, suggesting the function of Rb phosphorylation as a key cell cycle regulator in adipocyte lineage commitment and differentiation [47]. In addition, it is known that p38 activation can mediate an increase in the phosphorylation of Rb [48]. Therefore, our data on Rb phosphorylation are consistent with the observed activation of p38MAPK pathway.

Our approach to assess KGF role in adipogenic differentiation took in consideration also the evaluation of neutral lipids. De novo lipid biosynthesis is controlled by sterol regulatory element-binding proteins (SREBPs), which are not only generally activated in response to changes in intracellular and membrane levels of fatty acids and cholesterol but also strongly induced during adipogenic differentiation, since cholesterol accumulation is required very early during adipogenesis, in preparation for triglyceride synthesis and lipid droplet formation [49]. Moreover, previous works demonstrated a role of KGF in lipid metabolism, both in vitro on a pulmonary epithelial cancer cell line [22] and in vivo in a rat model [50]. In agreement with these data and with our initial hypothesis, we were able to demonstrate a KGF-dependent increase in neutral lipids (cholesterol, cholesterol esters, and tryglicerides), suggestive of a preparative step to initiate adipogenesis. Moreover, we observed that KGF treatment during adipogenesis was able to increase cell differentiation, determined as increased amount of oil red O stained lipid droplets, increased amount of mature adipocytes expressing FABP4 protein and higher expression of FABP4 mRNA in KGF-treated cells with respect to untreated cells. However, we were able to detect only a slight increase in PPAR*γ* expression in KGF-treated cells at the end of differentiation. Since PPAR*γ* upregulation is a late event in adipogenesis, we can hypothesize that in the advanced steps of this process, the effect of exogenous KGF might be partially masked by the upregulation of endogenous KGF that occur during differentiation.

It is known that FGFR2 gene is subjected to an alternative splicing to obtain the FGFR2-IIIc isoform, generally expressed in mesenchymal cells, or the FGFR2-IIIb isoform (also called KGFR), preferentially found in epithelial cells. Importantly, the only study reporting an effect of KGF on adipose cells failed to detect the expression of FGFR2-IIIb, the only known receptor that supports KGF signaling [21], suggesting that the effects of KGF on preadipocytes might be mediated through an unknown receptor rather than FGFR2-IIIb. Such hypothesis is also proposed by another study indicating KGF proliferative effect on endothelial cells derived from small vessels, which do not express FGFR2-IIIb [51]. However, since further studies demonstrated the expression of FGFR2-IIIb in vascular smooth muscle cells [52], we decided to assess FGFR2-IIIb expression in human ASCs. Our data demonstrated for the first time that ASCs, despite their mesenchymal origin, express a low amount of FGFR2-IIIb transcript and protein. Nevertheless, the open question was to determine if such FGFR2-IIIb was able to function even if expressed to a limited extent. To this aim, we took into account the activation of ERK, the main signaling pathway downstream KGF. Silencing of FGFR2 expression by means of small interfering RNA did not determine any variation of ERK phosphorylation, thus allowing us to conclude that FGFR2-IIIb is not responsible for KGF-mediated signaling pathway activation in ASCs. Such conclusion is supported by the known efficacy of FGFR2-IIIb silencing in the reduction of KGF effects on silenced cells, as previously demonstrated by our group [33].

Exploring the hypothesis of a nonclassical KGF receptor, we focused on NRP1, since it is indicated as a potential KGF interactor. Coimmunoprecipitation experiments allowed us to demonstrate that NRP1 is able to bind KGF in ASCs. Moreover, besides its role as receptor for class 3 semaphorins, NRP1 is also considered a coreceptor for some growth factors, such as VEGF or PDGF. Most data indicate that it lacks a defined signaling activity, but recent evidence obtained in melanoma cells has suggested that NRP1 is able to activate VEGF-mediated signal transduction pathways also independently of VEGFR-2 expression [25]. Consistent with this data, the results of our study show that the expression of NRP1 is necessary and sufficient for KGF-mediated activation of ERK, p38, and Akt pathways, thus indicating this molecule as a previously unrecognized nonclassical receptor for KGF. Interestingly, we found a differential expression of FGFR2 and NRP1 upon KGF treatment in time course experiments (see Figure S1). In particular, NRP1 downregulation was found to be less consistent than that of FGFR2, and limited to 15–30 min of KGF treatment. However, it is known that ligand binding should not necessarily induce receptor degradation. In fact, also for FGFR2 itself, it has been previously demonstrated that the receptor can have an alternative fate depending on the ligand, since it is ubiquitinated and degraded after KGF treatment but not after FGF10 treatment, probably due to a different regulation of receptor endocytic transport [53]. In the future, it will be interesting to investigate NRP1 intracellular fate upon treatment with KGF.

The evaluation of FGFR2-IIIb and NRP1 expression levels during adipogenesis represent a further confirmation of this hypothesis. In fact, we observed that endogenous KGF levels in differentiating ASCs increased in the first step of adipogenesis (days 1 and 2, see Figure 2), but at this time point, FGFR2-IIIb expression was still at basal levels, corresponding to a very low amount of protein (see Figure 6). The consistent amount of NRP1 protein in undifferentiated ASCs (see Figure 8(b)) and its further increase in the first days of adipogenesis (see Figure 10) support our hypothesis that NRP1 might mediate FGF7 activities during clonal expansion of ASCs, while FGFR2-IIIb mediates FGF7 action subsequently (maybe in association with NRP1 as a coreceptor). Of course, to definitely validate this working model, our future work will be dedicated to perform stable knockdown of FGFR2-IIIb and NRP1 in ASCs subjected to adipogenic differentiation.

## 5. Conclusions

Our study aimed to assess the role of KGF in human adipogenesis, in terms of proliferation and cell signaling activation. We believe that the discovery of an increase in KGF expression during adipogenic differentiation could contribute to a better understanding of the molecular bases of human adipogenesis, with potential implications for the therapeutical application of ASCs for regenerative medicine, as well as for the introduction of novel therapeutic strategies for obesity and other disorders related to alteration of lipid metabolism. Future work will be dedicated to better assess the role of KGF in adipogenesis, by means of approaches involving knockdown of KGF protein expression in ASCs and subsequent evaluation of adipogenic differentiation.

Moreover, the discovery of the role of NRP1, a widely expressed molecule, as a nonclassical KGF receptor indicates that its effects might be not limited to the epithelial tissues. Therefore, a deeper investigation of the specific contribution of the canonical receptor FGFR2-IIIb and the alternative receptor NRP1 to KGF signaling in different cellular contexts could contribute to better understand a number of physiological and pathological processes involving various tissues and organs.

## Figures and Tables

**Table 1:** List of primers used for the expression analysis of NRP1 by RT-PCR and custom primers/probes used for the expression analysis of FGFR2-IIIb and FGFR2-IIIc by qRT-PCR.

Gene	Forward primer sequence	Reverse primer sequence	Reporter sequence
NRP1	cctgaatgttcccagaactacacaa	caacatcagggaatccatccc	—
FGFR2-IIIb	ggctctgttcaatgtgaccga	gttggcctgccctatataattgga	ttccccagcatccgcc
FGFR2-IIIc	cacggacaaagagattgaggttct	ccgccaagcacgtatattcc	ccagcgtcctcaaaag

## Supplementary Materials

Supplementary Materials Supplemental Figure S1: Western blot analysis of the expression of FGFR2-IIIb and NRP1 in ASCs untreated or treated with KGF for 5′, 15′, 30′, 1 h, 2 h, and 3 h. Blotting with anti-tubulin antibody served as loading control. The images are representative of at least three independent experiments. The intensity of the bands was evaluated by densitometric analysis; the values from a representative experiment were normalized, expressed as fold increase with respect to the untreated sample (CTRL) and reported as a graph. Error bars represent standard deviations. ^∗∗^ *P* < 0.005 and ^∗∗∗^ *P* < 0.0005.
