# Supplementary figures and images for: Neuropilin 1 Mediates Keratinocyte Growth Factor Signaling in Adipose-Derived Stem Cells: Potential Involvement in Adipogenesis
Source: Stem Cells Int. 2018 Feb 25;2018:1075156. doi: 10.1155/2018/1075156 (PMC5845512; doi:10.1155/2018/1075156)

## Slide 1
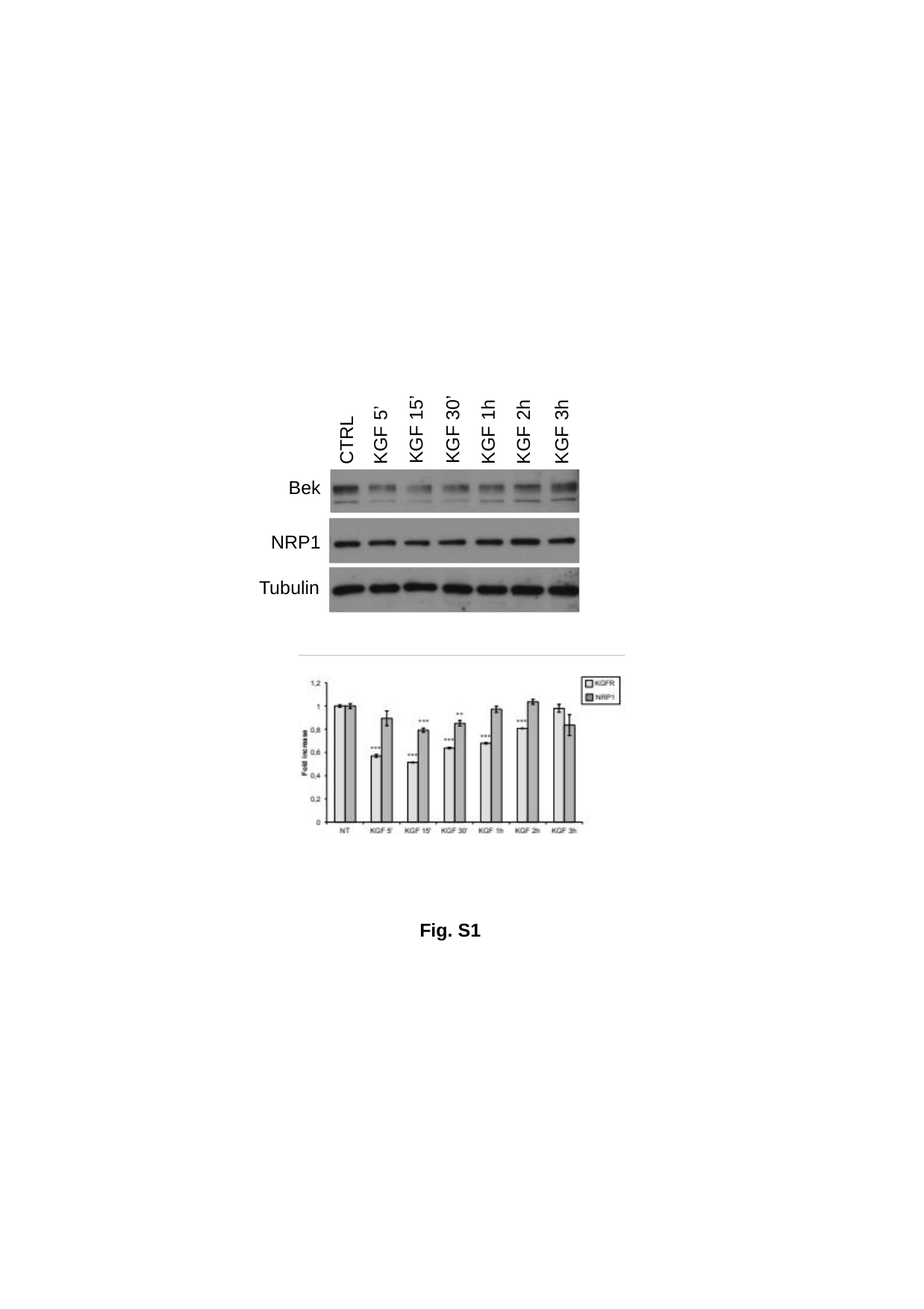

KGF 15’
KGF 30’
KGF 5’
KGF 1h
KGF 2h
KGF 3h
CTRL
Bek
NRP1
Tubulin
Fig. S1

Supplement: Supplementary Materials — Supplemental Figure S1: Western blot analysis of the expression of FGFR2-IIIb and NRP1 in ASCs untreated or treated with KGF for 5′, 15′, 30′, 1 h, 2 h, and 3 h. Blotting with anti-tubulin antibody served as loading control. The images are representative of at least three independent experiments. The intensity of the bands was evaluated by densitometric analysis; the values from a representative experiment were normalized, expressed as fold increase with respect to the untreated sample (CTRL) and reported as a graph. Error bars represent standard deviations. ∗∗ P < 0.005 and ∗∗∗ P < 0.0005. [file 1075156.f1.pptx]
